# “*Healthcare should be the same for everyone*”: perceived inequities in therapeutic trajectories of adult patients with lung cancer in Chile, a qualitative study

**DOI:** 10.3389/fpubh.2023.1228304

**Published:** 2023-08-16

**Authors:** Carla Campaña, Báltica Cabieses, Alexandra Obach, Francisca Vezzani

**Affiliations:** ^1^Centre of Global Intercultural Health (CeSGI) ICIM, Universidad del Desarrollo, Santiago, Chile; ^2^Programa de Doctorado en Ciencias e Innovación en Medicina (DCIM), Universidad del Desarrollo, Santiago, Chile; ^3^Center for Cancer Prevention and Control (CECAN), ANID FONDAP 152220002, Santiago, Chile

**Keywords:** therapeutic trajectory, lung cancer, social determinants of health, inequity, voice of patients

## Abstract

**Background:**

Globally, it has been reported that different social determinants of health affect health outcomes in lung cancer (LC). Research on the therapeutic trajectories of patients (TTP) is a novel field for identifying barriers and facilitators in health. The objective of this study was to reveal perceived differences in TTP with LC in Chile according to selected social determinants of health (SDH) and the experiences of patients, health professionals, and civil society leaders.

**Methods:**

This is a qualitative paradigm, one case-study design. Online semi-structured interviews were conducted with patients with LC, health professionals, and civil society leaders. The strategies for the recruitment process included social networks, civil society organizations, health professionals, and the snowball technique. A thematic analysis was carried out.

**Results:**

Selected SDH impact LC's TTP in Chile, particularly concerning health system access, health services, information, and patient navigation experiences. The analysis of the experiences of the participants allowed us to identify barriers related to the selected SDH in three stages of the TTP: initiation, examinations, and diagnosis and treatment. Individuals with limited education, those residing outside the capital, women, and those in the public health system encountered more barriers throughout their TTP.

**Discussion:**

Study findings suggest that being a woman with low education, from the public health system, and not from the capital might represent one of the most powerful intersections for experiencing barriers to effective healthcare in LC in Chile. It is necessary to monitor the TTP from an SDH perspective to guarantee the rights of access, opportunity, quality, and financial protection.

## Introduction

Worldwide, lung cancer (LC) is the second most diagnosed (11.4%) and the first in mortality, responsible for 1.8 million deaths according to the World Health Organization and Global Cancer Observatory (GLOBOCAN) (1, 2). LC mortality has a heterogeneous distribution in the population according to the social determinants of health (SDH) model. Some SDH that have been identified that can influence health trajectories and outcomes are being a woman (3, 4) having a low socioeconomic and educational level (5), type of health insurance (6), and place of residence (7).

In Chile, LC is fifth in incidence (7.3%) and first in mortality (12.4%) (8), and is recognized as a significant public health problem globally. Since 2019, LC has been incorporated into the law on explicit health guarantees in Chile (AUGE/GES). This law establishes the maximum waiting time for receiving care in the suspicion, diagnosis, and treatment stages. Furthermore, it guarantees access to high-quality services for all citizens regardless of age, sex, socioeconomic status, or health insurance (9). Regarding health insurance, Chile has a segmented system divided into public and private. Public insurance is the National Health Fund (FONASA), with 75% of the population covered, including those lacking resources and low education (9). The private system has Social Security Institutions (ISAPRES) as insurance (9). The service provider network is not integrated and generates barriers for patients across many health conditions and health needs, including cancer (10).

Research on SDH in LC in Chile is scarce; however, different mortality patterns are identified, for example, a rise in the crude mortality rate in women (11, 12), and an increase in mortality rate as the years of schooling decrease, even after adjusting by age and sex (13). A previous research reported that the general mortality from cancer in people with a low educational level doubles that of people with a university level (14). Regarding the place of residence, differences have been identified in the risk of dying from cancer according to the region of the country; in LC, the highest risk of dying is in the country's northern regions (12). This difference may be related to barriers to effective care (15).

A novel research approach to therapeutic trajectories of patients (TTP) contributes to a deeper understanding of patient experiences and identifies barriers and facilitators to accessing effective care (15). TTP has been defined as the complex and dynamic path a person—and their significant ones—follow in search of solutions to their health problems, including clinical assistance, treatment, and rehabilitation (16). This concept incorporates the multiplicity of needs and experiences that a person interacts with the health system (17) and the processes carried out by the health system to provide quality care (18); furthermore, TTP addresses the different stages of the disease process (beginning, diagnosis, treatment, and following), shedding special light on the voices of patients, significant ones, and other relevant actors like healthcare teams (18, 19).

There is relevant international literature on TTP, indicating that cancer is extensively studied in this research area (17), specifically through the patient navigation model (20). This model defines patient navigation (PN) as the support and guidance offered to individuals with abnormal screening tests or a new cancer diagnosis to access the cancer care system and overcome barriers (21). PN is currently used as a strategy to optimize healthcare for subgroups of cancer patients that face more barriers and, therefore, less access to healthcare (20, 22, 23). Studies on the impact of PN programs in cancer have reported a significant reduction in time to diagnosis and treatment initiation (24) and a reduction in inequities in access to healthcare (25). For this study, PN and TTP are used as synonyms.

Previous research in Chile on the SDH of LC has revealed significant differences in cancer incidence and mortality (3–7). However, how the TTP of LC patients might be influenced by different SDH models is heavily understudied. This article aims to reveal perceived differences in TTP of adult LC patients in Chile according to selected SDH: gender, educational level, region of residence, and type of health, according to the experience of patients, health professionals, and civil society leaders. These results complement a previous study by the researchers (26). Considering the impact of PN programs to decrease inequities in cancer, delving into the influence of the SDH on the TTP of cancer patients in Chile is essential to seek mechanisms that might explain these differences and to propose novel solutions for health system improvement in Chile and other countries.

## Methods

### Study design

We conducted a qualitative study with an exploratory approach (27) that allowed us to understand a less-known phenomenon, such as the TTP and SDH in LC in Chile, to begin our knowledge of the object of study. The study followed a case-study design, which enables an in-depth exploration of the phenomena in their naturally occurring context, involving the study of a bounded system (or case) within a contemporary setting through detailed, in-depth data collection (28). A case study was defined as the experience of the TTP of adults with LC in Chile with a focus on the influence of selected SDH in these trajectories. The study design followed the COREQ criteria (Supplementary Table 3).

### Participants

The number of participants was defined based on theoretical and feasibility criteria (27). At the beginning of the study, theoretical sampling establishes the profile of the study participants (29). The sampling units were threesome: (i) LC patients, (ii) health professionals who treat LC patients, and (iii) civil society leaders linked to LC. Given the focus on the complexity of the phenomena studied, qualitative research works with a few cases to deepen the meaning of the object of study, understanding reality through methods and techniques that produce narrative data (27). The number of participants was 18 patients, 8 health professionals (public and private health), and 1 civil society leader (total *n* = 27). The inclusion criteria for each group of participants are in Table 1. The exclusion criteria for all participants were a physical or mental condition limiting the person's ability to participate voluntarily in the study. The participants' decision-making capacity was assessed using the MacArthur criteria (30) during the informed consent process, ensuring their comprehension of the research project's information and implications. This encompassed awareness of potential effects, reasoning ability in decision-making, comparing alternatives and evaluating consequences, and freedom to express their choice in participating. Saturation of the information was assessed in the following dimensions: general TTP (beginning, diagnosis, and treatment), barriers, facilitators, needs, quality of care, and overall experience. After interim data analysis was conducted, information saturation was observed for all main dimensions of interest, and hence, we did not add participants to the sample size.

**Table 1:** Inclusion criteria for patients, health professionals, and civil society representatives.

**Patients**	**Health professionals**	**Civil society leader**
**Inclusion criteria**		
(i) Over 18 years of age. (ii) Being treated for lung cancer in the healthcare public or private system in Chile. (iii) Having Internet access or telephone to participate in the interview.	(i) Working in the Chilean public or private health system. (ii) Being a specialist in any health profession in lung cancer. (iii) Having Internet access to participate in the interview.	(i) Over 18 years of age. (ii) Participating with an active role in civil society organizations related to lung cancer. (iii) Having Internet access to participate in the interview.

All participants were characterized demographically (Table 2). To address the absence of a civil society leader dedicated exclusively to LC in Chile, we interviewed a civil society leader from Argentina.

**Table 2:** Socio-demographic characteristics of PARTICIPANTS.

**Identification code**	**Health system**	**Gender**	**Age**	**Education**	**Region of residence**
**Patients**					
P1F_PUBLIC	Public	Female	69	Incomplete	Metropolitan
P2F_PUBLIC	Public	Female	59	Incomplete	Metropolitan
P3F_PUBLIC	Public	Female	63	Complete	Metropolitan
P4M_PUBLIC	Public	Male	71	Complete	Other region (northern)
P5M_PUBLIC	Public	Male	77	Complete	Metropolitan
P6M_PUBLIC	Public	Male	65	Complete	Metropolitan
P7F_PUBLIC	Public	Female	76	Incomplete	Metropolitan
P8M_PUBLIC	Public	Male	63	Complete	Metropolitan
P9F_PUBLIC	Public	Female	56	Incomplete	Metropolitan
P10F_PUBLIC	Public	Female	80	Incomplete	Metropolitan
P11F_PRIVATE	Private	Female	64	Complete	Other region (southern)
P12M_PUBLIC	Public	Male	76	Complete	Other region (southern)
P13M_PUBLIC	Public	Male	67	Incomplete	Metropolitan
P14F_PUBLIC	Public	Female	66	Incomplete	Other region (northern)
P15M_PUBLIC	Public	Male	60	Complete	Other region (northern)
P16M_PUBLIC	Private	Male	73	Complete	Other region (southern)
P17M_PUBLIC	Public	Male	64	Incomplete	Other region (northern)
P18M_PUBLIC	Public	Male	76	Incomplete	Metropolitan
**Health professionals**					
**Identification code**	**Health system work**	**Gender**	**Age**	**Medical specialty**	**Region of work**
M1H_PUBLIC	Public	Male	63	Oncologist	Metropolitan and northern
M2H_PUBLIC	Public	Male	37	Oncologist	Metropolitan
M3H_PUBLIC/PRIVATE	Public/Private	Male	49	Thorax surgeon	Other region (southern)
M4H_PUBLIC	Public	Male	35	Oncologist	Metropolitan
M5H_PRIVATE	Private	Male	54	Hematooncologist	Metropolitan
M6H_PUBLIC	Public	Female	-	Oncologist	Other region (northern)
EU1_PUBLIC	Public	Female	31	Nurse	Metropolitan
EU2_PUBLIC/PRIVATE	Public/Private	Female	38	Nurse	Metropolitan
**Civil society leader**					
**Identification code**	**Work**	**Gender**	**Age**	**Specialty**	**Country**
Soc. Leader	Patient foundation	Male	52	Economist and public policy	Argentina

For patient participants, complete education is 13 or more years of schooling, and incomplete education is < 13 years.

### Recruitment and data collection

The recruitment process was carried out between October 2021 and March 2022. Strategies for recruiting patients included social networks, civil society organizations, health professionals, and the snowball technique (31). Interested participants provided contact information (telephone or email). Later, they were contacted by the study coordinator. In the case of agreeing to participate, the coordinator schedules the virtual, semi-structured interview. For health professionals and civil society leaders, recruitment was based on a mapping of relevant actors conducted by the research team. They were contacted via email and telephone, following a similar process as other participants. Virtual semi-structured interviews (Zoom, WhatsApp, and Meet) were conducted due to the COVID-19 pandemic, allowing access to patients in different regions using a pre-defined script based on the study's objectives (27). Sociodemographic data (age, sex, health insurance, region, and education) were collected at the beginning.

The semi-structured interview script included the following dimensions: (i) general therapeutic trajectory; (ii) barriers to healthcare; (iii) healthcare facilitators; (iv) health needs throughout the therapeutic trajectory; (v) quality of care; and (vi) overall evaluation of the experience. Table 3 presents the areas explored and the questions used for each group of participants. Interviews were facilitated by two trained staff, audio-recorded, and securely stored on a personal computer. During the interviews, patients could request to be accompanied by a family member.

**Table 3:** Dimensions, codes, and questions of interview and analysis.

**Participants**	**Dimension**	**Code**	**Questions**
Patients	General experience	General health status	How is your current state of health?
	Therapeutic trajectory	General experience	How would you describe your therapeutic trajectory?
		First symptoms and access	What were the first symptoms? How did you detect them? Why did you decide to consult?
		Diagnosis process	How was the diagnostic process? Can you mention all the details that you remember?
		Treatment process	How was the treatment process? Can you mention all the details that you remember? How do you feel that the treatment has affected your life in general?
	Barriers and facilitators	Barriers	What barriers did you face to achieve the entire process of diagnosis, treatment and recovery, if applicable, for this disease? At what point in your therapeutic trajectory did you face those barriers?
		Facilitators	What facilitators did you face to achieve the entire process of diagnosis, treatment and recovery, if applicable, for this disease? At what point in your therapeutic trajectory did you face those facilitators?
Health professionals	Therapeutic trajectory	General information	How would you describe the therapeutic trajectory in the health system?
		Times in therapeutic trajectory	Do you identify differences in the time of diagnosis or treatment between health institutions (i.e., public, private, or region)?
	Barriers and facilitators	Barriers	What are the barriers to the therapeutic trajectory of the patient? At what stage of the therapeutic trajectory do these barriers appear? What barriers are from the health system, and what barriers are from the user or his family?
		Facilitator	What are the facilitators in the therapeutic trajectory of the patient? At what stage of the therapeutic trajectory appear these facilitators? What facilitators are from the health system, and what barriers are from the user or his family?
Civil society leader	Therapeutic trajectory	General information	How would you describe the experience of living with lung cancer? How would you describe the therapeutic trajectory of patients with lung cancer? Please consider the activities the patients had to carry out in the health system for diagnosis, treatment, and recovery.
		Diagnosis process	How is the diagnostic process?
		Treatment process	How is the treatment process?
	Barriers and facilitators	Barriers	What barriers do patients with lung cancer have to face to achieve the entire process of diagnosis, treatment and recovery, if applicable, for this disease? At what moment of the therapeutic trajectory do those barriers appear?
		Facilitator	What facilitators do patients with lung cancer have to face to achieve the entire process of diagnosis, treatment, and recovery, if applicable, for this disease? At what moment of the therapeutic trajectory do those facilitators appear?

### Data analysis

Based on audio records, a verbatim transcription of all the anonymized interviews into Microsoft Word was made. Two members of the research team confirmed the transcripts of the interviews. Each interview was assigned a unique code to ensure participant information and confidentiality. Deductive thematic analysis was carried out manually using an interview matrix, a qualitative method that allows for identifying thematic patterns from the data collected (27). Information was organized based on categories according to pre-defined dimensions identified in the literature on therapeutic trajectories in cancer. In each category, codes were identified that were accompanied by participant quotes. This analysis delves into each TTP dimension from the SDH lens based on the following additional dimensions: educational level, gender, region of residence, and type of health insurance. Each TTP dimension was described in depth based on codes that specifically described each stage of the navigation process for adult LC patients (Table 2). For publication purposes, the research team translated the textual citations from Spanish to English and ensured accurate interpretation.

### Rigor

The study applied the following rigorous criteria: triangulation of responses from participants (patients, health professionals, and civil society leaders) and reflexivity notes were considered (28, 32). Triangulation contrasted and compared participant groups' findings to obtain a more precise and comprehensive understanding of the studied phenomenon. Reflexivity entailed recording researchers' reflections, emotions, observations, and methodological decisions during interviews, enabling critical self-evaluation and regular research approach reviews with the investigation team.

## Results

The selected SDH had an influence mainly on access to the health system, access to health services, access to information, and navigation in the health system. The results identify three specific moments where the SDH was influencing the TTP: (i) beginning, (ii) examinations, and (iii) diagnosis and treatment. Additionally, the results revealed that gender consistently influences the entire therapeutic trajectory. Results are summarized in Table 4.

**Table 4:** Barriers and facilitators in lung cancer patient's trajectory from a SDH perspective.

**SDH involved**	**Therapeutic trajectory stage**	**Barriers**	**Facilitators**
Education level	Beginning	Delay in initial consultation.	Knowledge about health status. Empowerment in medical-patient relationship. Knowledge about health guarantees.
	Diagnosis	Access to scanners or biopsies. Unclear information about characteristics of diagnosis.	
	Treatment	Access to treatment.	
Health Insurance	Beginning	Physician without experience in lung cancer. Unavailable exam in the public network. High costs for imaging exam. Bureaucracy. Difficult navigation in the health system for administrative and management process. Not integrated public health network. Not integrated public and private health network.	Earlier access to a physician. Access to a requests tests to asses health status.
	Diagnosis	Access to tests. Unclear information about characteristics of diagnosis. Physician-patient relation.	
	Treatment	Access to treatment	
Place of residence	Beginning	Quality of the exams. Access to testing hours. Centralization of health institutions and specialists.	
	Diagnosis	Travel to get a diagnosis.	Regional center public institutions.
	Treatment	Access to treatment. Lack of infrastructure of local health institutions.	Regional center public institutions.
Gender	Beginning-Diagnosis-Treatment	Female. Loneliness. Changes of living roles.	Male. Company. Motivation.

### SDH influence at the beginning

Participants perceive that an incomplete educational level acts as a barrier that impacts the time people decide to make an initial consultation for symptoms or signs, such as cough, tiredness, and fatigue. Unfortunately, these symptoms are known by patients and are associated with colds or other causes, and they do not provide immediate warning signs to patients, delaying the first cancer consultation. Patients with complete education show a better understanding of their health status and knowledge of medical benefits. Moreover, empowerment is observed in the medical-patient relationship, which allows them to express their requirements and be considered in medical decisions.

“*Probably access is much easier for a patient in the private area, usually due to socioeconomic issues, it is a patient who has a higher level of schooling and education and that also favors earlier diagnoses and in people with low resources and low educational level there is not much awareness that it is necessary to consult” (EU1M-PUBLIC/PRIVATE)*

The type of health insurance also generates differences at the beginning of TTP. Patients with private insurance who consult for respiratory symptoms can access a physician much sooner, who then requests tests to assess their health status, including imaging tests. This evaluation allows for early suspicions of LC and subsequent diagnosis.

“*In August 2012, I had a very, very strong cold with a lot of decay, unusual for the colds I always had, and I went to a doctor here in the region and [SIC] sent me to do many tests, like a complete check-up, and among those tests, there was a chest scan...there it appeared, when the test was done, a nodule in the right lung” (P11M-PRIVATE)*

Contrastingly, the request for imaging tests for patients with public insurance with similar symptoms is not as frequent. Therefore, some patients with public insurance make their first consultation in the private system due to delays and mistrust of the public health system. After this first consultation, these patients receive a medical order to have an imaging exam (CT, MRI, or RX); however, they cannot afford to pay out-of-pocket for them and therefore return to the public system. If patients undertake their first consultation in primary care, they can access only a general physician without experience with LC and receive a diagnosis of other diseases.

“*I decided not [SIC] go directly to a, to..., to the Cesfam (*primary healthcare) *that are so tedious because they attend you and they never give you the result of anything, they make you wait in excess” (P13H-PUBLIC)*

“*Primary Health Care physicians don't know that lung cancer exists, they do not diagnosis.” (M1H-PUBLIC)*

When suspicion of LC is established, people with public insurance experience new barriers related to the bureaucracy of the public system. Administrative and management barriers at the primary level of care are due to ignorance of how to navigate the system, long waiting lists, and a lack of equipment, to mention the most frequent ones. Sometimes, patients with public insurance are referred by primary healthcare physicians to perform specific exams in health institutions with a higher level of complexity or in the private system due to their unavailability in the public network. This situation produces worry and confusion because the system is not integrated, and patients must learn and understand how to access the new health institutions. Patient organizations contribute to addressing these situations.

“*Occasionally I get confused in so many places… It's like having a horrible confusion, because if from one hospital they send you to another, from another… now they send me to the private clinic, they send me from one place to another, the truth is that I don't understand” (P13H-PUBLIC)*

“*The biggest problem, I would say, is that in general, the health professional doesn't know who to refer to, because the patient must be referred to more than one specialist” (M2H PUBLIC)*

“*Support to, to be able to resolve some critical issues that in the system appears, The patient in particular, due to his condition, is very difficult for him to overcome” (Soc. Leader)*

### SDH influence in testing exams

Despite the fact that all interviewed patients with incomplete secondary education reported having public insurance, only some developed the entire TTP in the public system, largely due to delayed access to testing for the diagnosis of cancer. In order to afford private care, significant out-of-pocket spending was required, yet they chose it due to a lack of trust and long waiting lists in the public system. The mixed navigation of a proportion of patients with public health insurance becomes, at some point, a barrier of its own because the public and private systems are not sufficiently interconnected.

“*The scanners, the magnetic resonance scanning, all those exams, the most difficult thing was to get the hours for that day... we didn't have the conditions to pay privately” (P7M-PUBLIC)*

“*FONASA Patients arrive with their notification from a private practice that a doctor who requested a scan and found a suspicious nodule in neoplasia and they arrive, they are Fonasa patients and have to be treated here. A way to bring them was created because it was a mess” (M3H-PUBLIC/PRIVATE)*

Patients who fully navigate their TTP in the public system can also be referred to the private system when the waiting time for examinations or diagnosis is longer than the legal guarantee. In these cases, having a complete education is perceived as a facilitator because it allows the person to have more abilities to access and understand available information like what is guaranteed by law and how to demand its fulfillment.

“*The deadline expired. It was a month for them to give me attention and it was not like that, and I asked the second provider and I was referred, well, I also asked for a place that was... that was more advanced and they sent me to Santiago” (P15H-PUBLIC)*

People with public or private insurance who do not reside in the Metropolitan region describe barriers related to the quality of the exams carried out in regions and lower access to testing hours. In addition, the patient and family must cover the expense of travel, accommodations, and food to get to the metropolitan region or another region nearby where they can take their examinations. In this case, the centralization of health institutions and specialists is a critical barrier for patients who live in other regions despite having health insurance.

“*The first thing that even the doctors tell you…go to Santiago, and one arrives in Santiago with the regional exams, they don't take them into account and they take them again…so it's all a waste of money for people” (P11M-PRIVATE)*

### SDH influence on diagnosis and treatment

Diagnosis and staging of LC are mainly obtained using scanners or biopsies. Differences in opportunities for these tests are perceived among participants based on education and health insurance. In addition, differences are recognized in how people express cancer information. People with an incomplete education provide information according to size, and people with a complete education provide information about size and stage. These differences may be related to how health professionals communicate with people with an incomplete education, which is unclear and possibly generates false expectations.

“*They did a scan and mmm..., and they saw it was a tiny tumor” (P9M-PUBLIC)*.

“*I know that I am in stage four, terminal” (P3M-PUBLIC)*

During treatment, it is also possible to identify perceived differences in TTP with LC related to education, health insurance, and region of residence. Patients with public insurance with incomplete education perceived having complex treatments, such as chemotherapy, radiotherapy, surgeries, and immunotherapy. This is perceived to be associated with more severe stages of the condition at diagnosis for patients with these characteristics. The same perception is reported by healthcare teams. Patients with complete education and public health insurance were perceived to have fewer treatments, for example, surgeries or chemotherapy. Experiences of access to treatment of patients with public insurance are varied, with patients who access the treatment and others who are still waiting. In the case of patients who are not from metropolitan region, as in the other stages of the TTP, the barriers are associated with access due to the lack of infrastructure of local health institutions.

“*For a long time with immunotherapy, then the immunotherapy stopped giving results, back to chemo and then they diagnosed the brain metastasis started with radiotherapy, ten sessions and also combined with chemo, that is, it was quite a bomb”[SIC] (P10M-PUBLIC)*

“*In private centers what they find is stage 1, that is, there are small nodules...the patient with early stage lung cancer is potentially curable” (M1H-PUBLIC)*

### Gender as a cross-cutting SDH during TTP of LC patients

Gender is perceived to influence the TTP and LC profoundly. Male patients are generally accompanied by a woman throughout the whole TTP, either the wife, the daughter, or the granddaughter. In the case of female patients, the beginnings of TTP are lonely, and the motivation to recover is associated with their caregiving role. In addition, having to leave these roles due to the progress of the disease generates discomfort and anger.

“*Look, at us, my husband was diagnosed, he was diagnosed because I took him to the doctor, because he had a lot of cough” (P13H-PUBLIC)*

“*I didn't tell my children, I went to the hospital by myself, that's how it was” (P2M-PUBLIC)*

## Discussion

This study explored through qualitative research the perceived influence of SDH during the TTP with LC in Chile, according to patients, healthcare teams, and leaders of LC organizations. The qualitative approach to the study problem has been recognized as relevant for addressing health inequalities (33, 34). Qualitative studies have focused on investigating the causes of delays in LC diagnosis.

Our study allows us to identify that people with incomplete educational levels (under 13 years), residents of regions outside the metropolitan area, women, and those belonging to the public health system face more barriers and fewer facilitators during their TTP. Herb et al. (35) identified that barriers during TTP can impact timely access to healthcare and health outcomes. In the case of Chile, the differences identified in our study related to SDH can influence the time to access and health outcomes, but more studies are necessary.

Other studies identified delays in the patient's decision to seek the initial consultation related to the type of symptoms, knowledge, fear, and cultural patterns (34, 36). Our study recognizes education level and the healthcare system as the leading social determinants impacting the decision to consult and being associated with less empowerment of patients in their relationship with the healthcare system and providers. These results are similar to those obtained by Sayani et al. (37), and Saab et al. (38), making it possible to recognize patterns of inequities in LC at a local and global level. In this context, primary healthcare is relevant to address this inequity; unfortunately, our study results identified important barriers in primary healthcare related to TTP, including professionals lacking experience or knowledge in suspecting LC, delays in test management and results, and administrative processes that complicate patient navigation.

Although the results of our study reveal that educational level and the health system generate the most significant number of barriers, place of residence and gender have been less studied and also generated barriers during TTP. Our study suggests that living outside of the Metropolitan region of the country is also a negative SDH for the TTP of LC patients, despite the patient's health insurance. This situation might be secondary to a lack of sanitary infrastructure for diagnosis and treatment. This reality deepens socioeconomic health inequities in the country due to long waiting lists, late diagnosis, and personal/family travel costs and accommodation to other regions to accelerate the time to diagnosis and treatment (39, 40).

Interestingly, gender was perceived as a deep, structural, and cross-cutting SDH of the TTP with LC in Chile. Barriers related to conventional gender stereotypes such as housekeeping and caregiving (41, 42) were constantly described throughout the TTP. The therapeutic trajectories of male patients were generally perceived to be accompanied by a female family member. On the contrary, the therapeutic trajectories of female patients were vastly described as lonely. Loneliness can influence individuals' motivations and relationships with the health system during the entire navigation (42). From an intersectional lens (43, 44), being a woman with low educational attainment in the public health system and not a resident of the metropolitan region might represent one of the most powerful intersections for experiencing barriers to effective healthcare in LC in Chile.

To overcome unequal therapeutic trajectories in LC, Chile must invest and implement specific strategies to involve patients in their healthcare (45) and in a patient-centered care model (46) to secure effective trajectories for these patients, including subgroups that are left behind based on evidence-informed SDH (47–49).

This is the first study in Chile to describe the influence of selected SDH in the TTP of LC from different actors and using a qualitative perspective. Hence, the study highlights the voice and experience of patients as well as health professionals and civil society leaders. The qualitative approach allowed us to explore emerging specific aspects related to the existing inequities based on a SDH lens.

This study has both strengths and limitations. Limitations are as follows: (i) limited representation of multiple and diverse existing subgroups in the country (ethnicities, regions of residence, etc.) and (ii) the difficulty of accessing patients with more severe stages of LC, which made it impossible to analyze the following stage. Despite the limitations, we believe this study contributes with a unique and unprecedented vision around the perceived influences of several SDH on the TTP of LC patients. Future studies could consider expanding sampling strategies to other population groups, adding other SDH such as age, type of occupation, and marital status, as well as mixed methods to integrate qualitative with quantitative data for a better understanding of this phenomenon.

Study findings suggest that selected SDH might play an essential, interactive, and changing role throughout the TTP in LC in Chile. To guarantee the rights of access, opportunity, quality, and financial protection, it is necessary to monitor the TTP from a SDH perspective.

## Data availability statement

The original contributions presented in the study are included in the article/Supplementary material, further inquiries can be directed to the corresponding author.

## Ethics statement

The studies involving human participants were reviewed and approved by the Ethics Committee of the Faculty of Medicine of Universidad del Desarrollo. The participants provided their written online informed consent to participate in this study.

## Author contributions

CC, BC, and AO: conceptualization and study design. CC and FV: data collection and data analysis. CC and BC: manuscript writing. AO and FV: review and editing. All authors contributed to the article and approved the submitted version.
