# Supplementary material for: “Healthcare should be the same for everyone”: perceived inequities in therapeutic trajectories of adult patients with lung cancer in Chile, a qualitative study
Source: Front Public Health. 2023 Aug 16;11:1228304. doi: 10.3389/fpubh.2023.1228304 (PMC10468573; doi:10.3389/fpubh.2023.1228304)
Supplement: Supplementary file 1 [file Table_1.DOCX]

Supplementary Material

***"Health care should be the same for everyone”:* Perceived Inequities in therapeutic trajectories of adult patients with lung cancer in Chile, a qualitative study.**

Carla Campaña, Báltica Cabieses^*^, Alexandra Obach, Francisca Vezzani

*** Correspondence:** Corresponding Author: bcabieses@udd.cl

# Supplementary Table

**Supplementary Table 1:** Interview Guide for in-depth interviews with Adults with the experience of lung cancer in Chile.

| **INTERVIEW GUIDE FOR PATIENTS** |
| --- |
| **Instructions to the interviewer** |
| 1. Apply the interview script only once the online informed consent has been signed.  2. Have a backup audio recorder handy if the main one fails.  3. Inform to the participant the approximate duration of the interview, between 45 and 60 minutes.  3. Observe the participant's emotional state at all times, stopping whenever necessary and checking if he/she wants to continue participating. |
| **Opening sentence** |
| Welcome participants and introduce yourself.  Explain the purpose and process of the in-depth interview.  Explain the presence and purpose of recording equipment. |
| **Demographic data from the interviewee** |
| Ask about name, age, sex, gender, region of residence, education level, profession/activity, health system |
| **Discussion guidelines** |
| ***Therapeutic trajectory***  General questions about health condition and current health status of patients.  How would you describe your therapeutic trajectory (that is, each of the activities he had to carry out in the health system for his diagnosis, treatment, and recovery if he had already reached that point)? Let's start with the first symptoms... which were? How do you detect them?  How you agreed and consulted, what made you consult... when decide to consult and why?  How was the diagnostic process experience... what emotions marked the beginning of your disease?  How was/is the treatment process experience, can you tell me with all the detail that you remember.  Have you experience some adverse effects or change on your quality of life, which are this, can you tell me and explain how this impact in your live? |
| ***Health care barriers and facilitators***  In your opinion, what are the barriers that you are experienced and achieve during the entire process of diagnosis, treatment, and recovery, if applicable, for the lung cancer?  At what point did you face that barrier? how was that experience? what did you learn from it? how did you overcome?,  Now, about the facilitators that you have to experience during the entire process of diagnosis, treatment and recovery, if applicable, for this disease, can you name some of these facilitators? how was your experience with these facilitators? what you think about theses facilitators? |
| **INTERVIEW GUIDE FOR HEALTH PROFESSIONALS** |
| **Instructions to the interviewer** |
| 1. Apply the interview script only once the online informed consent has been signed.  2. Have a backup audio recorder handy if the main one fails.  3. Inform to the participant the approximate duration of the interview, between 45 and 60 minutes.  4. Observe the participant's emotional state at all times, stopping whenever necessary and checking if he/she wants to continue participating. |
| **Opening sentence** |
| Welcome participants and introduce yourself.  Explain the purpose and process of the in-depth interview.  Explain the presence and purpose of recording equipment. |
| **Demographic data from the interviewee** |
| Ask about name, age, sex, gender, region of residence, education level, profession/activity, health system |
| **Discussion guidelines** |
| ***Therapeutic trajectory***  General information: In your opinion as health professional of patients with lung cancer, How is the experience of live with lung cancer?  How would you describe the therapeutic trajectory of patients with lung cancer?  Please consider the activities the patients had to carry out in the health system for diagnosis, treatment, and recovery.  How is the start?, which are the most frequently symptoms or signs?  How is the process to get the diagnosis of lung cancer? How live this moment the patients? How the health system supports this process? How you think about this process as a health professional.  How is the process to get a treatment for lung cancer? How live this moment the patients? How the health system supports this process? How you think about this process as a health professional?. Which are some adverse effects or change in quality of life of patients with lung cancer? can you tell me and explain how this impact the live of patients? |
| ***Health care barriers and facilitators***  In your opinion, what are the barriers that the patients with lung cancer must experience and achieve during the diagnosis and treatment?  At what point patients face these barriers? how is that experience? how these barriers became least presences for other patients?  Now, about the facilitators, can you describe some facilitators that help to the patients with lung cancer in the therapeutic trajectory?, can you recognized these facilitators in diagnosis or treatment stage? how is the experience of patients with these facilitators? how these facilitators can became presences for other patients? |
| **INTERVIEW GUIDE FOR CIVIL SOCIETY LEADER** |
| **Instructions to the interviewer** |
| 1.Apply the interview script only once the online informed consent has been signed.  2. Have a backup audio recorder handy if the main one fails.  3. Inform to the participant the approximate duration of the interview, between 45 and 60 minutes.  4. Observe the participant's emotional state at all times, stopping whenever necessary and checking if he/she wants to continue participating. |
| 1. Opening sentence |
| Welcome participants and introduce yourself.  Explain the purpose and process of the in-depth interview.   1. Explain the presence and purpose of recording equipment. |
| **Demographic data from the interviewee** |
| Ask about name, age, sex, gender, region of residence, education level, profession/activity, health system |
| **Discussion guidelines** |
| ***Therapeutic trajectory***  General information: In your opinion as leader of patients with lung cancer, How is the experience of live with lung cancer?  How would you describe the therapeutic trajectory of patients with lung cancer?  Please consider the activities the patients had to carry out in the health system for diagnosis, treatment, and recovery.  How is the start?, which are the most frequently symptoms or signs?  How is the process to get the diagnosis of lung cancer? How live this moment the patients? How the health system supports this process? How you think about this process as a leader of patients with lung cancer.  How is the process to get a treatment for lung cancer? How live this moment the patients? How the health system supports this process? How you think about this process as a leader of patients with lung cancer. Which are some some adverse effects or change in quality of life of patients with lung cancer? can you tell me and explain how this impact the live of patients? |
| ***Health care barriers and facilitators***  In your opinion, what are the barriers that the patients with lung cancer must experience and achieve during the diagnosis and treatment?  At what point patients face these barriers? how is that experience? how these barriers became least presences for other patients?  Now, about the facilitators, can you describe some facilitators that help to the patients with lung cancer in the therapeutic trajectory?, can you recognized these facilitators in diagnosis or treatment stage? how is the experience of patients with these facilitators? how these facilitators can became presences for other patients? |

**Supplementary Table 2:** Additional quotes for each stage of therapeutic trajectory

| **Beginning of TTP** | *"They diagnosed him because I took him to the doctor, because he had too much cough... at the moment he started to cough, he, he was throwing up blood..."(P13H-PUBLIC)* |
| --- | --- |
|  | *“I had pain in my spine and when I had a medical consultation, I asked for a scan and it was taken on September 4 of the year that passed from…twenty…2021, (The doctor) did not give importance to it, he did give importance to it, but I knew that because of the pain, something was not going well, and when I had the consultation afterwards carrying out the tests, it appeared, in the lung... in a lung, first in the left and then it went to the right" (P15H-PUBLIC)* |
|  | *"It was fatigue more than anything, that was what gave us the warning sign to take her to the doctor and the doctor ordered a scan...(PCTE) when they just found the cancer I lived sitting" (P10M-PUBLIC)* |
|  | *"It was just a cough, and in fact we took her because she had that cough...according to the doctor at the clinic, she had the cancer since five years ago" (P14M-PUBLIC)* |
|  | *"...March, April he didn't want to go to the doctor, after that he coughed a lot, due to that cough we took him to a medical center in May-June to be evaluated by a doctor and that doctor sent him to have an exam , it was a..., a resonance... we have done everything in the private system... because one knows that in the public system one... one dies waiting, why say it in other words?" (P4H-PUBLIC)* |
|  | *"The general relationship between the vision of the PHC and the hospital system is distant" (EU2M-PUBLIC/PRIVATE)* |
| **Exam Stage** | *"If you don't move, if you don't move and you stay there, "laying in the eggs", as they say, saying "no, they haven't called me yet, I've been waiting for a year", and What happened?, you died, waiting for them to call you and it's not a lie….if I don't move I'm not going to have surgery and I'm going to die” (p2m-public)* |
|  | *"I took him to the Cesfam, at the Cesfam he was treated by a very good doctor who told me "look, the first thing we are going to do is an exam", he sent him to me, he gave me the order and I had to pay to do that exam that It cost me fifty something, now, and that exam shows that he has lung cancer..." (P13H-PUBLIC)* |
|  | *"It's terrible, terrible, an exam one hundred thousand pesos, an MRI one hundred eighty thousand pesos... my children helped me, my children "mommy don't go around taking money", they put some money and together we did the money and I did the PET” (P2M-PUBLIC)* |
|  | *“The trip to santiago was financed by a car from the municipality, he offered a car because I moved there, since I was here in xxx I moved there and there we got a car to go to stgo to take the exam… the rest was financed by my daughter, all my children helped me with money, with gasoline, with money for the trips for everything, they also made some collections there" (P17H-PUBLIC)* |
|  | *“I understand that it is due to a cost issue, but it is very unequal, because a patient lives longer if they have complementary insurance and are treated here in Los Andes than if they are treated in a public hospital in the southern sector of Santiago and that is like this” (M5H-PRIVATE)* |
|  | *Everything is concentrated in santiago, in regions efforts are made but in the end we all try to get to santiago... I was wasting time for 2 years... no one covers travel, accommodation, tickets... If I had not had the conditions to do so, the facilities to do it between the ISAPRE and the complementary insurance... I don't know until what stage I would have reached..." (P11M-PRIVATE)* |
| **Diagnosis** | *And that biopsy showed that he has a... large tumor that measures more than seven centimeters and is scaly" (P4H-PUBLIC)* |
|  | *"Mine was a small cancer because I arrived on time." (P2M-PUBLIC)* |
|  | *"He had a 2.5 nodule, a year..., then he had..., he grew something three centimeters" (P7M-PUBLIC)* |
| **Treatment** | *"The nurse told me look, she told me the truth, you are, you were lucky, she told me, if you had surgery, she told me, super fast" (P2M-PUBLIC)* |
|  | *“I am undergoing radiotherapy, there are thirty sessions” (P7M-PÚBLICO-EI-RM)* |
|  | *"I thought at first that it was for treatment, maybe, for the problem I had and not, it's to prevent it from spreading to other organs" (P12H-PUBLIC)* |
|  | *"He has never been without treatment, let's say, we cannot say that they have neglected this issue, no, nothing, he has always been treated" (P10M-PUBLIC)* |
|  | *"So much paperwork to do when you're sick, it shouldn't be like that, it should be more expeditious, faster... I've had to wait a long time to be attended or to get treatment" (P12H-PUBLIC)* |
|  | *“We are hoping that he will soon have treatment that is not palliative. And if it is palliative, at least it should be something more than Paracetamol” (P13H-PUBLIC)* |
|  | *“That doctor operated me, and I was hospitalized for eight days, that is, three days and then I continued monitoring with him for two, three years” (P16H-PUBLIC)* |

**
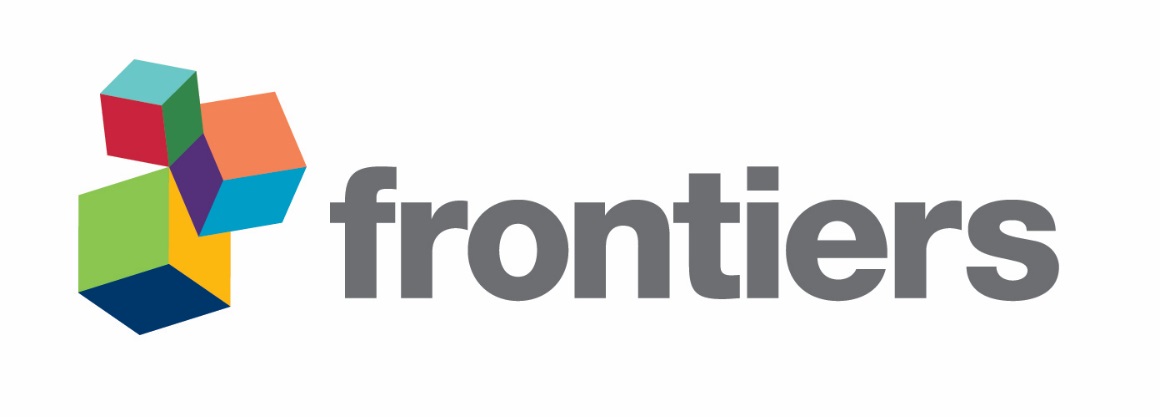
**

**Supplementary Table 3:** CORE-Q checklist of the research *"Health care should be the same for everyone”:* Perceived Inequities in therapeutic trajectories of adult patients with lung cancer in Chile, a qualitative study.

| Item | Topic | Guide Question | Descriptions | Report on (section/table) |
| --- | --- | --- | --- | --- |
| **Domain 1: Research team and reflexivity** | | | | |
| *Personal characteristics* | | | | |
| 1 | Interviewer/facilitator | Wich author/s conducted the interview? | Data from in-depth interviews are presented in this manuscript. | Section 2.3 |
| 2 | Credentials | What were the researcher’s credentials? | We describe the credentials of the researchers who participated in this study. | **Supplementary Table 4** |
| 3 | Occupation | What was their occupation at the time of the study? | The occupations of the researchers on the study team are describe in the manuscripts and in Supplementary Material | **Manuscript section 8 Supplementary Table 4** |
| 4 | Gender | Was the researcher male or female? | The genders and initials of all the researchers are described in Supplementary Material | **Supplementary Table 4** |
| 5 | Experience and training | What experience or training did the researcher have? | Experience and training of each researcher is describe in supplementary Material | **Supplementary Table 4** |
| *Relationship with participants* | | | | |
| 6 | Relationship established | Was a relationship established prior to study commencement? | The researchers informed to all potential participants the study objectives and right to decline participation or withdraw consent at any research stage were informed to potential participants. All participants have the opportunity prior the study to make all questions to the researcher by mail or phone. All participants provided an online informed consent to be part of the study. | Manuscript, Section 2.3 and 6 |
| 7 | Participant knowledge of the interviewer | What did the participants know about the researcher? (e.g., personal goals, reasons for doing the research) | Potential participants knows the name of the main researcher, place of work and reason for doing the research. Also, they were informed of the reason and importance of this research. | Manuscript, Section 6 |
| 8 | Interviewer characteristics | What characteristics were reported about the interviewer/facilitator? E.g., bias, assumptions, reasons and interest in the research topic? | Potential participants knows the name of the interviewer, place of work and reason for doing the research. Also, they were informed of the reason and importance of this research. | Manuscript, Section 6 |
| **Domain 2: Study design** | | | | |
| *Theoretical framework* | | | | |
| 9 | Methodological orientation and theory | What methodological orientation was stated to underpin the study? E.g., grounded theory, discourse analysis, ethnography, phenomenology, content analysis. | We employed the thematic analysis in this study | Manuscript, section 2.4 |
| *Participant selection* | | | | |
| 10 | Sampling | How were participants selected? E.g., purposive, convenience, consecutive, snowball. | We used a convenience sample for selected study participants depending on theoretical criterias establishes, and availability, willingness to participate. | Manuscript, section 2.2 |
| 11 | Method of approach | How were participants approached? E.g., face-to-face, telephone, mail, email. | Participants were mainly recruited after they indicate a phone or mail to contact for the interest to participate in the study.  Research team used social networks, civil society organizations, health professionals, and snowball technique to recruited participants. | Manuscript, section 2.3 |
| 12 | Sample size | How many participants were in the study? | There was a total of 27 participants in the study. | Manuscript, section 2.2; and table 3. |
| 13 | Non-participation | How many people refused to participate or dropped out? Reasons? | Participation in the in-depth interviews was voluntary and no participant refused to participate. | N/A |
| *Setting* | | | | |
| 14 | Setting of data collection | Where was the data collected? E.g., home, clinic, workplace. | For the pandemic context the data collected was online. | Manuscript, section 2.3 |
| 15 | Presence of non-participant | Was anyone else present besides the participants and researchers? | Patients could request to be accompanied by a family member. | Manuscript, section 2.3 |
| 16 | Description of sample | What are the important characteristics of the sample? E.g., demographic data, date. | Participants’ sociodemographic data are described on the manuscript. | Manuscript, section 3.1; and table 3. |
| *Data collection* | | | | |
| 17 | Interview guide | Were questions, prompts, guides provided by the authors? Was it pilot tested? | The Interview guide is provided by the research team in supplementary material. | **Manuscript table 2, Supplementary Table 1** |
| 18 | Repeat interviews | Were repeat interviews carried out? If yes, how many? | There were no repeat interviews carried out in this study | N/A |
| 19 | Audio/visual recording | Did the research use audio or visual recording to collect the data? | Recorders were used to audio-record interviews, which were later transcribed verbatim, translated into English, and checked backwards for accuracy for the research team. | Manuscript, section 2.4 |
| 20 | Field notes | Were field notes made during and/or after the interview or focus group? | n/a | n/a |
| 21 | Duration | What was the duration of the interview or focus group? | The interviews lasted between 45-60 minutes | **Supplementary Table 1** |
| 22 | Data saturation | Was data saturation discussed? | Saturation of the information was assessed after interim data analysis by the research team. | Manuscript, section 2.2 |
| 23 | Transcripts returned | Were transcripts returned to participants for comment and/or correction? | The transcripts of the interviews were confirmed by more than one member of the research team. | Manuscript, section 2.4 |
| *Data analysis* | | | | |
| 24 | Number of data coders | How many data coders coded the data? | The manuscript contain the information about the coded data for this study | Manuscript, section 2.3 and table 2 |
| 25 | Description of the coding tree | Did authors provide a description of the coding tree or codebook? | The manuscript contains the information about the codebook for this study | Manuscript, table 2 |
| 26 | Derivation of themes | Were themes identified in advance or derived from the data? | Themes identified in advance according to the existing literature | Manuscript, section 2.3 |
| 27 | Software | What software, if applicable, was used to manage data | N/A | N/A |
| 28 | Participant checking | Did participants provide feedback on the findings? | Participants received via e-mail a resume for the findings. Participants did not give feedback. | S/I |
| *Reporting* | | | | |
| 29 | Quotations presented | Were participant quotations presented to illustrate the themes/findings? Was each quotation identified? e.g., participant number. | Representative quotes and the respective code number are embedded within the text. | Manuscript, section 3, and supplementary table 2 |
| 30 | Data and findings consistent | Was there consistency between the data presented and the findings? | We demonstrate consistency between data presented in the results section and the interpretation of findings in the Discussion section. | Manuscript, section 3 and 4 |
| 31 | Clarity of major themes | Were major themes clearly presented in the findings? | We presented all major themes according the objective and method of the study. | Manuscript, section 3 |
| 32 | Clarity of minor themes | Is there a description of diverse cases or discussion of minor themes? | We provide a diversity of quotes in the manuscript and in supplementary material. | Manuscript, section 3, and supplementary table 2 |

**Supplementary Table 4:** Research Team Characteristics and Qualifications

| **Author** | **Characteristics and Qualifications** |
| --- | --- |
| CC | Female with a Master of Management in Primary Health Care, and PhD© for the Doctor in Sciences and Innovation in Medicine program. Qualitative, multi-methods, and epidemiology research training. Her research interests include cancer, rare diseases, inequities, global health, and patient participation. |
| BC | Female Nurse-midwife (2002, PUC Chile), diploma in university teaching (2004, PUC Chile), Master in Epidemiology (2008, PUC Chile) and PhD in Health Sciences (mention in social epidemiology) from the University of York, England (2011). Professor of social epidemiology and director of the Center of Global Intercultural Health at the Faculty of Medicine Universidad del Desarrollo. Visiting scholar at the Department of Health Sciences at the University of York. Member of Lancet Migration for Latin America, board member of the Chilean network of research on health and migration RECHISAM. Former Vice-President of the Chilean Society of Epidemiology between the years 2014-2017. National and international consultant of health equity, health of migrants and implementation science in socially and culturally diverse communities. Advisor to WHO, PAHO, the Ministry of Health and the Ministry of Social Development of Chile, and to various public and private institutions. Research lines: social inequities in health (2005 to date), health of international migrants (2008 to date) and participation of patients in decision-making on health coverage (2016 to date). Editor of 8 academic books and over 20 publicly available research reports and policy briefs. Participation in more than 60 research projects in Chile and abroad and has more than 170 scientific publications. |
| AO | Female Social Anthropologist. She has a master's degree in Gender and Cultural Studies from the University of Chile, and a doctorate in Social and Cultural Anthropology from the University of Barcelona. Associate Professor and Executive Director of the Center for Global Intercultural Health (CeSGI), Universidad del Desarrollo. She is an expert in health, gender and intercultural issues. Research focuses on health anthropology, adolescent and youth sexual and reproductive health, patient participation in health decision-making, and qualitative methodologies. Principal investigator of various research projects, including: Fonis Project #SA15I20040; Fonis Project #SA19I0091; Fondecyt #11190701. |
| FV | Female, Social Anthropologist, MSc (c) in Public Health (Universidad de Chile). Diploma in Quantitative Methodologies (Universidad Diego Portales), and diploma in Formulation and Evaluation of Social Projects focused on the territory and the community (Pontificia Universidad Católica de Chile). Researcher in Center for Global Intercultural Health (CeSGI), ICIM, Universidad del Desarrollo. Research interests include anthropology and health, public health, inequities, patient participation, cancer and rare diseases. |
